# The Insula: A Stimulating Island of the Brain

**DOI:** 10.3390/brainsci11111533

**Published:** 2021-11-19

**Authors:** Inès Rachidi, Lorella Minotti, Guillaume Martin, Dominique Hoffmann, Julien Bastin, Olivier David, Philippe Kahane

**Affiliations:** 1 CHU Grenoble Alpes, 38000 Grenoble, France; LMinotti@chu-grenoble.fr (L.M.); GMartin@chu-grenoble.fr (G.M.); DHoffmann@chu-grenoble.fr (D.H.); PKahane@chu-grenoble.fr (P.K.); 2 Univ. Grenoble Alpes, Inserm, U1216, Grenoble Institut Neurosciences, 38000 Grenoble, France; julien.bastin@univ-grenoble-alpes.fr (J.B.); Olivier.David@inserm.fr (O.D.)

**Keywords:** insula, epilepsy, SEEG, direct cortical stimulation, cortico-cortical-evoked potentials

## Abstract

Direct cortical stimulation (DCS) in epilepsy surgery patients has a long history of functional brain mapping and seizure triggering. Here, we review its findings when applied to the insula in order to map the insular functions, evaluate its local and distant connections, and trigger seizures. Clinical responses to insular DCS are frequent and diverse, showing a partial segregation with spatial overlap, including a posterior somatosensory, auditory, and vestibular part, a central olfactory-gustatory region, and an anterior visceral and cognitive-emotional portion. The study of cortico-cortical evoked potentials (CCEPs) has shown that the anterior (resp. posterior) insula has a higher connectivity rate with itself than with the posterior (resp. anterior) insula, and that both the anterior and posterior insula are closely connected, notably between the homologous insular subdivisions. All insular gyri show extensive and complex ipsilateral and contralateral extra-insular connections, more anteriorly for the anterior insula and more posteriorly for the posterior insula. As a rule, CCEPs propagate first and with a higher probability around the insular DCS site, then to the homologous region, and later to more distal regions with fast cortico-cortical axonal conduction delays. Seizures elicited by insular DCS have rarely been specifically studied, but their rate does not seem to differ from those of other DCS studies. They are mainly provoked from the insular seizure onset zone but can also be triggered by stimulating intra- and extra-insular early propagation zones. Overall, in line with the neuroimaging studies, insular DCS studies converge on the view that the insula is a multimodal functional hub with a fast propagation of information, whose organization helps understand where insular seizures start and how they propagate.

## 1. Introduction

**Figure 1:**
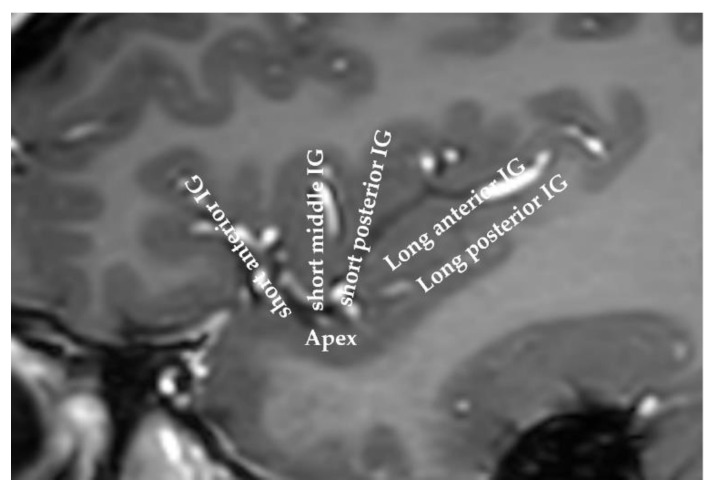
MRI of a human insula. The anatomy of the insula can be viewed on this sagittal slice**.** The insula is classically divided into the anterior insula and the posterior insula, which are separated by the central insular sulcus. IG: insular gyrus.

The insula, or “the fifth lobe of the brain”, was first described in 1809 by the anatomist Johann Christian Reil [1]. This small area of the cortex is buried in the sylvian fissure, covering less than two percent of the total cortical surface area. This lobe is split by the central insular sulcus into two parts: the anterior insula and the posterior insula. The anterior insula comprises three gyri (anterior, middle, and short gyri), while the posterior insula encompasses the anterior and posterior long gyri (Figure 1).

Compared with other superficial cortices, access to the insula during surgical procedures was difficult for several decades due to its deep localization and the rich vascular network running in the lateral fissure separating the insula from the frontal, parietal, and temporal opercula. Thus, though the macroscopic, cytoarchitectonic, and myeloarchitectonic characteristics of the insula were described in the first half of the twentieth century [2,3], the functions of this “island” remained quite unexplored for a long time, especially in epilepsy surgery patients. In the early fifties, however, Penfield et al. paved the way for the discovery of some of insular properties through direct electrical cortical stimulations (DCSs) of the insula during surgical procedures in epileptic patients [4,5]. Nonetheless, the low benefit/risk ratio of insular lobe resection in epilepsy surgery patients [6] explained why the insular lobe was not investigated further for many years.

In the early 2000s, Isnard et al. rekindled interest for the insula in epilepsy surgery patients studied with stereotactic intracerebral EEG (SEEG) recordings [7,8], and the insula has since become a thorough subject of study in epilepsy. In parallel, the advent of neuroimaging techniques allowed the progressive discovery of its wide array of functions and high connectivity to other brain areas, leading to increasing interest in this structure [9,10,11,12,13,14]. In this context, clinical seizure analysis as well as insular DCS studies performed during SEEG procedures also provided important new information that helped unveil some properties of this small but highly functional and densely connected cortex.

We will briefly review the findings of DCS of the insula in epilepsy surgery candidates subjected to intracranial EEG recordings regarding functional mapping, connectivity, and seizure elicitation.

## 2. Direct Cortical Stimulations (DCSs): General Considerations

Since the pioneering works of Penfield and Jasper [15], DCS has appeared as a powerful technique to study network mechanisms in epilepsy, including functional mapping of brain functions, estimation of functional connectivity, assessment of cortical excitability, and elicitation of seizures [16]. In the clinical setting, however, DCS is mainly performed with the twofold aim to reproduce the patient’s aura or typical complete seizure and to map cortical functions to assist in tailoring surgery [17].

**Figure 2:**
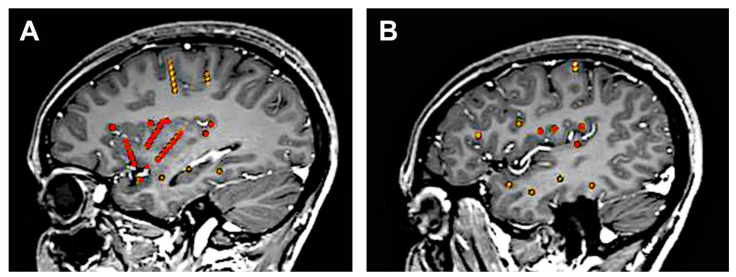
Typical SEEG investigation of the insula**.** This 25-year-old female had seizures that clinically strongly suggested insulo-opercular involvement without clear scalp EEG lateralization. Bilateral asymmetric SEEG implantation was performed but only for the left side, which is shown. The insulo-opercular region was covered by (**A**) three oblique electrodes exploring the anterior and posterior short gyri and the anterior long gyrus of the insula, as well as by (**B**) several lateral orthogonal electrodes that reached the insula through the supra-sylvian and temporal opercula. Additional electrodes also explored the temporal region and the motor cortex. Insular electrodes are depicted in red, while other electrodes are depicted in yellow. EEG: electroencephalography; SEEG: stereo-electroencephalography.

DCS can be applied during different types of invasive evaluation (e.g., intra-operative electrocorticography, extra-operative subdural, or depth electrodes). Among these different types, the SEEG method is especially well suited in the context of insular investigation, since it uses multi-contact intracerebral electrodes that penetrate the brain and therefore give direct access to deep-seated structures that cannot be recorded using subdural grids or strips. The ideal coverage of the insula can thus be obtained by combining a lateral orthogonal trajectory through the frontoparietal and temporal opercula [7] with an oblique approach through the frontal and parietal cortices to allow a larger insular sampling [18] (Figure 2). Combined depth and subdural electrodes [19,20] or hybrid operculo-insular electrodes [21] can be also used to investigate the insulo-opercular complex.

Stimulations during SEEG studies are classically conducted over one or a few days under continuous video EEG monitoring during sessions that may last from one to few hours. Typically, trains of a biphasic (alternating polarity) stimulus at a low (1–10 Hz) or high (50–60 Hz) frequency are applied between contiguous electrode contacts (bipolar stimulation) with stepwise increasing intensities until clinical responses or after-discharges are obtained and, according to the parameters, proven to produce no structural damage [22]. Depending on the objectives of the study, the type of stimulation (low or high frequency), the duration of the trains (around 5 s at 50 Hz and 40 s at 1 Hz), and the current intensity (0.2–8.0 mA) will vary depending on the level of cortical excitability and the degree of the clinical manifestations expected by stimulating a given structure.

When proceeding this way, DCS permits a fair degree of selectivity of the response with a high degree of localization, since the stimulus is delivered in bipolar mode through adjacent contacts only 2–5 mm away from each other (center to center) and because the stimulated contacts are not located on the pia-arachnoid but inside the cortex, which avoids a current “leak” through the cerebrospinal fluid [23]. Obviously, such a “phrenological” approach cannot account for the complexity of brain (dys)functions, which are currently viewed as underlain by large-scale networks (i.e., distributed groups of interconnected and synchronized neurons) rather than isolated functional areas [24,25]. Additionally, how DCS modulates brain activity so that behavioral responses are altered or promoted still remains to be elucidated. However, despite some reservations, DCS remains a unique method to provide information on some of the key structures involved in various perceptual and behavioral phenomena, whether physiological or pathological [26].

## 3. Functional Mapping of the Insula

Since the first visceral and somatosensory maps of insular stimulation provided by Penfield and Faulk in 1955 [5], and thanks to the growing development of SEEG, several insular stimulation studies came out in the last two decades [8,27,28,29,30,31,32], the main results of which are summarized in Table 1. Additional DCS studies dedicated to specific clinical domains also provided new insights into how the insula is involved in brain processing of various modalities such as pain [33,34], taste and smell [35], audition [36], vestibular functions [37], autonomic activity [38], and cognitive-affective behaviors [39,40,41].

All these studies were conducted using (sometimes not exclusively) high-frequency DCS, which has been found to be much more efficient than low-frequency DCS for eliciting clinical events [42]. Some studies included clinical responses that were recognized as identical to ictal clinical symptoms by the patients, which represented up to 22.3% (31/139) of the elicited signs [8]. Most of the studies, however, only considered clinical responses that did not resemble a patient’s epileptic aura and were not associated with an after-discharge, thus minimizing the possibility that the elicited signs were produced from an epileptogenic cortex where plastic changes may have occurred in response to repeated seizures. Still, most of the DCSs seemed to provoke only one single clinical event, which might suggest a functional selectivity of the stimulated area. Whether this effect results from a local or remote effect of the electrical current delivered remains debatable [34], but this supports the idea that the DCS sites sit in critical nodes of the neural network underlying a specific brain process.

Overall, it appears that the insula can be viewed as an eloquent structure, considering the high rate of clinical responses elicited by its stimulation (75% in a recent review [43]). The respective proportion of elicited signs varied across studies, possibly due to variations in electrode placement and the stimulation parameters. The vast majority of the clinical responses consisted of somatosensory sensations, which were most often contralateral to the DCS site and were frequently reported as paresthesiae-like, thermal, and painful sensations [8,28,30,31,32]. However, as already noticed by Penfield and Faulk [5], visceral manifestations—of which constrictive pharyngo-laryngeal and thoracic sensations are the most typical—were also quite frequent and even dominated the scene in some studies [27,29]. Other responses belonging to all sensory domains (e.g., gustatory, olfactory, auditory, and vestibular)—with the striking exception of vision—were much less frequent but were almost consistently observed across multiple studies. This supports the idea of multimodal sensory processing within the insula. Speech disturbances—the mechanism of which remains unclear—and motor manifestations—of which some could be related to the close vicinity of the pyramidal tract—were also reported.

Though the localization of a given type of clinical response varied across different studies, with a spatial overlap between the responses, some degree of spatial segregation emerged from the DCS studies, with visceral functions located in the anterior part of the insula and somatosensory functions in its posterior portion. In particular, pain responses were densely observed in the postero-superior part of the insular cortex [34], with a possible somatotopic representation showing a face area rostral to the upper and lower limb areas [33]. Other sensory modalities also seemed to respect some kind of anatomical organization, although with much overlap: (1) gustatory (and much more rarely olfactory) sensations were mainly provoked dorsally in the middle and posterior insular short gyri [35] in a region which appeared more as a multimodal area involved in feeding behavior rather than a unimodal sensory integrative area [44]; (2) auditory responses were mainly triggered by DCS of the postero-inferior insula [8,28,29,32], of which some could possibly be due to a remote effect of the electric current on the Heschl gyrus, which is in a close vicinity of this part of the insula. We recently showed that the posterior insula was a key region for eliciting auditory hallucinations, while auditory illusions were evoked more anteriorly [36]. This agrees with a differentiated role played by the insula, depending on the level of complexity of the perception; and (3) vestibular responses were mainly elicited from the long insular gyri—mostly in the lower part—with rotatory and translational sensations being produced more posteriorly than other vestibular signs [37].

Interestingly, “silent” or “non-eloquent” responses, though infrequent, still account for one fourth of insular DCSs [43] and might represent an evaluation bias. Heart rate changes, for instance, were not so rare when specifically studied and carefully assessed using time–frequency analysis of RR variations (47% of 100 DCSs), showing equal bradycardia and tachycardia responses that were predominantly provoked by anterior and posterior DCSs, respectively [38]. Moreover, most of the “silent” DCSs have been observed during stimulation of the anterior insula, known to have an upper “cognitive” part and lower “emotional” part. Perceptual or behavioral changes due to DCS of the anterior insula are thus difficult to assess, considering the limited time frame that allows DCS and in the absence of specifically dedicated tasks. As a matter of fact, only a handful of DCS studies have been able to show changes in cognitive processes or in emotional states, including ecstatic sensations provoked by anterior dorsal insular stimulation [39], enhancement of anger recognition by anterior insular stimulation [40], and modulation of evoked emotional states, depending on the magnitude of the electrical current delivered within the anterior insula [41].

Despite some limitations, the DCS data were consistent with the functional imaging findings that revealed four functionally distinct insular regions in the human, with a posterior sensori-motor part, a central olfacto-gustatory region, a ventral-anterior social-emotional portion, and a dorsal-anterior cognitive pole [9]. They also substantiated the concept of a posterior-anterior gradient of intra-insular information processing, where interoceptive signals are integrated in the posterior and central insula together with salient environmental stimuli before being transmitted to the anterior insula, which in turn provides the substrate for high-level cognitive functions in parallel with the information coming from frontal and limbic structures [9,13,14]. Further interpretation of DCS effects needs new developments, such as the application of DCS during specific cognitive or emotional tasks [45] or the quantification of DCS-induced high-frequency activities outside the stimulated region to assess large-scale networks correlated with DCS clinical responses [36,46].

## 4. Functional Connectivity of the Insula

Since the early 1980s, a variety of studies in human and nonhuman primates have shown that the insular lobe has dense local intra-insular and extra-insular connections to the frontal, parietal, and temporal lobes as well as to the thalamus and basal ganglia [47,48,49,50]. More recently, diffusion-weighted MRI and fiber tractography have allowed for refining structural connectivity in humans, showing a rostro-caudal and dorso-ventral organizational pattern, with preferential connections of the anterior and posterior insula with the anterior and posterior brain regions, respectively [51]. This organizational pattern seems to follow, at least in part, some cytoarchitectonic rules, with the ventral anterior agranular sector of the insula being connected to the dysgranular or agranular cortical areas and the posterior granular sector of the insula being connected to granular areas [49].

Structural connectivity studies, though of particular relevance, do not provide information on the directionality or propagation latencies along large fibers, an issue that can be addressed by electrophysiology. In particular, such *functional* connectivity can be studied intracranially in humans by applying low-frequency DCSs to the cortical areas and recording the electrophysiological responses in distant connected structures, a method known as cortico-cortical evoked potentials (CCEPs) [52,53]. This methodology has been used to map cortical networks related to various brain processes such as language, motricity, or vision, as well as to study intra- or inter-hemispheric connectivity [52]. Following this line of research, a new method for studying CCEPs in patient populations was proposed to summarize the electrophysiological findings between patients by normalizing a patient’s brain MRI into a stereotactic space after automatic processing and a data quality check [54]. As part of the F-tract project (f-tract.eu, accessed on 13 April 2021), this approach was further developed in hundreds of patients, thus providing a probabilistic atlas of brain connectivity derived from several thousand stimulations run in many cortical areas [55,56,57].

**Figure 3:**
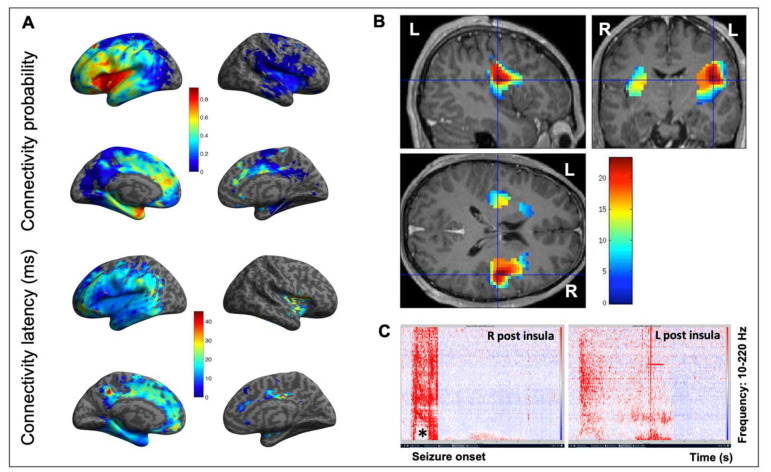
Connectivity of the insula. (**A**) Connectivity probability (upper part) and connectivity latency (lower part) maps obtained using CCEPs of the insula in 107 patients of the F-tract database (f-tract.eu) (unpublished data). The insula is part of a dense and widely extended network in which CCEPs propagate quickly, first with a higher probability around the insular DCS site and then to other cortical regions. (**B**) Epileptogenicity map of an SEEG-recorded spontaneous seizure, which evaluates the propensity of a brain region to generate significant fast activities (60–200 Hz) at seizure onset. This 21-year-old male patient was suffering from seizures characterized by a painful reflex left arm sensation followed by loss of contact, bilateral dystonic posturing of the upper limbs, hypermotor behavior, and postictal aphasia. Note how both insula are involved at seizure onset, though with a right-side predominance, which underlines how lateralization can be an issue in insular epilepsy due to the close and fast inter-insular connections. (**C**) Time frequency analysis at seizure onset of the same seizure, which confirms the simultaneous and asymmetric involvement of two homologous insular subregions (L and R postero-superior insula). Note how fast frequencies are suppressed (*) concurrently with the increase in high frequencies. L: left; R: right.

Overall, only a handful of studies have used CCEPs to specifically assess insular connectivity, the main results of which can be summarized as follows: (1) As a rule, CCEPs propagate first and with a higher probability around the insular DCS site, then to the homologous region, and later to more distal regions [55,57] (Figure 3). Areas with fast propagation (short peak latency responses) are usually those with the highest connectivity probability, meaning that the connectivity is likely to be direct [55]. (2) The intra-insular connectivity shows that the anterior insula has a higher connectivity rate with itself than with the posterior insula, and the same applies for the posterior insula [55]. However, when going into more detail, it appears that a weak connectivity was found between agranular and other insular cortices, while a rich connectivity was found between dysgranular and granular cortices, as well as within the insular subregions of a similar architectony [58]. (3) Both the anterior and posterior insulae are closely connected [55,59] (Figure 3), with a fast (8–24 ms) propagation time between the homotopic anterior insular parcels [60]. However, cross-connectivity between the insular cortices varies depending on the hemisphere or the stimulated gyrus [59], which can explain why some studies have failed to bring out interhemispheric connections [61]. (4) All insular subdivisions show extensive and complex ipsilateral and contralateral extra-insular connections [55,59], with connections of the left insula to language areas while the right insula is rather connected to cortices devoted to sensory, pain, vestibular, and saliency processing [59]. Extra-insular connectivity therefore shows rich connections with various brain regions, including the frontal, temporal, parietal, and even visual cortices [55,59,61,62]. These connections have been found to be reciprocal, with the highest connectivity rates observed with the perisylvian structures [61]. More recent data suggest the highest connectivity rates are to a large part of the frontal and temporal lobes and to the cingulate cortices as well [55]. The connectivity patterns, however, differ across the five insular gyri [59,61] with regionally specific organization, as the anterior insula tends to connect to more anterior areas and vice versa for the posterior insula [55,62].

The assessment of insular connectivity by studying CCEPs revealed dense and widely extended networks which roughly overlap with those identified by neuroimaging studies. In addition, it provided complementary data on the dynamical properties of the information flow within the insula and between the insulae and distant brain areas that would benefit from being merged with the structural data. Interestingly, a very recent study using a biologically informed modeling approach fitted to the early N1 component of CCEPs showed that almost all efferent and afferent insular connections were faster than 10 ms without exhibiting the distance-dependent lengthening of the cortico-cortical axonal conduction delays observed for the other brain regions [56]. Such a finding is in line with the idea that the insula is a hub which needs to integrate and process multimodal information quickly.

## 5. DCS-Elicited Insular Seizures

Although DCSs of the human epileptic brain have been in use for several decades to mimic the effects of an epileptic discharge in epilepsy surgery patients [15], only a few studies have investigated the relevance of DCS-induced seizures (DCS-S) in clinical practice. A review of 14 studies identified during a 30-year period showed that DCS-S were observed in 37–100% of patients undergoing an invasive evaluation with a large predominance of elicited auras, of which most were provoked from the temporal lobe or from the posterior cortex [63]. Complete seizures were less frequent and were observed in any location, though they were more frequently in the frontal lobe. Further additional studies showed on the one hand that DCS-S had a strong electrical and clinical concordance with spontaneous seizures [64] and, on the other hand, that DCS-S represented a positive predictive factor for seizure outcomes after surgery [65,66], especially when spontaneous seizures and DCS-S showed a high level of similarity [67].

**Figure 4:**
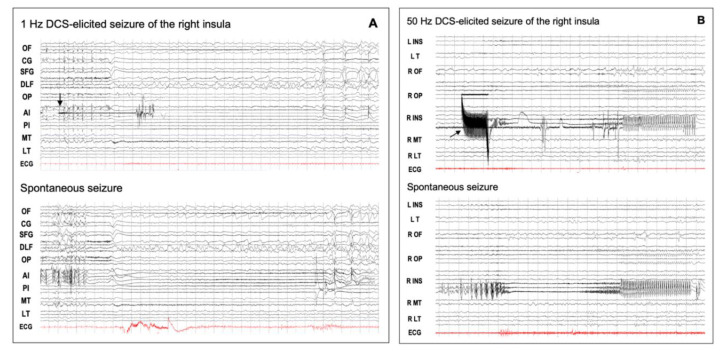
DCS-induced seizures. (**A**) Low-frequency (1 Hz) DCS-induced and spontaneous insular seizures. This 18-year-old male patient had seizures characterized by an explosive hypermotor behavior without any aura. From the start of stimulation (arrow), the elicited discharge was quite visible between the DCS artifacts, and its spatio-temporal organization was quite similar to that of the spontaneous seizure. OF: orbito-frontal cortex; CG: cingulate gyrus; SFG: superior frontal gyrus; DLF: dorsolateral frontal cortex; OP: opercular cortex; AI: anterior insula; PI: posterior insula; MT: mesio-temporal structures; LT: lateral temporal cortex; ECG: electrocardiogram. (**B**) High-frequency (50 Hz) DCS-induced and spontaneous insular seizures for the same patient as in Figure 2. The right postero-superior insular DCS (arrow) induced a seizure which showed the same time evolution and spatial organization as that of the spontaneous discharge, except that the initial part of the elicited discharge was masked by DCS artifacts. DCS: direct cortical stimulation; L: left; R: right: INS: insula; T: temporal lobe; OF: orbito-frontal cortex; OP: opercular region; MT: mesio-temporal structures; LT: lateral temporal cortex; ECG: electrocardiogram. These EEG recordings can be viewed in a larger size in the supplemental material (Figure S1).

No study, unfortunately, specifically dealt with insular DCS-S, and little information can be extracted from SEEG series of insular lobe epilepsy. Auras or complete electro-clinical seizures were shown to be elicited by DCSs of the insula in adults (9/12 cases [68]) as well as in children (2/8 [69]), a rate that did not differ from that of other DCS studies conducted by means of intracerebral electrodes [63]. High-frequency (50 Hz) DCSs seemed more prone than low-frequency DCSs (1 Hz) to elicit seizures [68], a finding already reported for other locations [42]. Low frequency DCSs, however, allow a better visualization of the SEEG recording during stimulation, a feature particularly interesting at the onset of insular DCS-S which, as spontaneous insular seizures, start focally with limited intra-insular spread [68] (Figure 4A,B). Interestingly, Singh et al. found that although DCS-S were mainly provoked from the insular seizure onset zone in a majority of the cases, some could also be triggered by stimulating the intra-insular propagation zone, as well as the extra-insular areas involved in early propagation of the seizures [68]. This latter aspect reinforces the idea that an epileptogenic zone behaves as a hierarchically organized network rather than a focus [70] which can be modulated by DCSs at any part of its primary organization.

## 6. Conclusions

The modulatory effects of DCS on cortical activity in epileptic patients is not well understood, and it depends on a variety of factors that include the stimulation parameters (which may produce either inhibitory or excitatory responses), the intrinsic neuronal activity (which may influence the threshold for cortical excitability and varies across cortical areas), and the eventual presence of an epileptogenic cortical environment (which may facilitate the neuronal response and its propagation). Despite these limitations, DCS data have provided, by accessing the cortex directly, important information on the many functional roles of the insular subregions and the multiconnected routes through which these functions are processed. Accordingly, they also brought forth helpful information to localize epileptic auras more accurately and to better understand the richness of insular epilepsy semiology.

## Figures and Tables

**Table 1:** Functional mapping studies of the insula. Percentages were rounded to the nearest whole number (downward when the fraction was less than 0.5 and upward when the fraction was 0.5 or greater). Note that the percentages do not total 100%, since one single DCS can produce several types of clinical responses simultaneously. NA: not available; N°: number; DCS: direct cortical stimulation; DCS (+): DCS eliciting clinical signs; freq: frequency; VisceroS: viscerosensitive; VisceroM: visceromotor; SomatoS: somatosensory. Neurovegetative symptoms were classified as visceromotor when not further specified.

Study [Reference]	N° of Patients	DCS freq (Hz)	N° of DCS Sites	N° of DCS (+) Sites	N° of DCS	DCS Eliciting Clinical Phenomena Not Recognized by the Patient as Part of the Seizure and Not Accompanied by an Afterdischarge										
						N°	VisceroS	VisceroM	SomatoS	Gustatory	Olfactory	Auditory	Vestibular	Speech	Motor	Others
Ostrowsky 2000 [27]	13	50–1	27	20	75	32	17 (53%)	5 (16%)	7 (22 %)	3 (9%)	1 (3%)	1 (3%)	0	3 (9%)	0	2 (6%)
Isnard 2004 [8]	50	50–1	144	125	139	108	34 (31%)	3 (3%)	58 (54%)	3 (3%)		14 (13 %)	5 (5%)	9 (8%)	0	6 (6%)
Nguyen 2009 [28]	9	50	36	32	96	NA	6%	0 %	62 %	6 %	0%	9 %	3%	3%	12%	0%
Afif 2010 [29]	25	50–1	25	22	179	67	28 (42%)		19 (28%)	0	0	3 (4%)	4 (6%)	8 (12%)	11 (16%)	3 (4%)
Pugnaghi 2011 [30]	61	50–1	165	NA	276	152	2 (1%)	2 (1 %)	105 (69%)	2 (1%)	0	12 (8%)	3 (2%)	9 (6%)	12 (8%)	2 (1%)
Stephani 2011 [31]	5	50	113	62	113	54	17 (31%)	0	30 (56%)	7 (13%)	0	0	0	0	0	0
Mazzola 2017 [32]	222	50	669	NA	669	550	82 (15%)	0	335 (61%)	15 (3%)	6 (1 %)	44 (8%)	41 (8 %)	27 (5%)	0	0

## Data Availability

Group analyses of cortico-cortical evoked potentials are available on the f-tract atlas (f-tract.eu/atlas/) which has been published in PerSCIDO (https://perscido.univ-grenoble-alpes.fr/datasets/DS331.

## Supplementary Materials

The following are available online at https://www.mdpi.com/article/10.3390/brainsci11111533/s1, Figure S1: DCS-induced seizures (This figure is larger EEG image of Figure 4 in the main text.)
