# Supplementary figures and images for: The Insula: A Stimulating Island of the Brain
Source: Brain Sci. 2021 Nov 19;11(11):1533. doi: 10.3390/brainsci11111533 (PMC8615692; doi:10.3390/brainsci11111533)

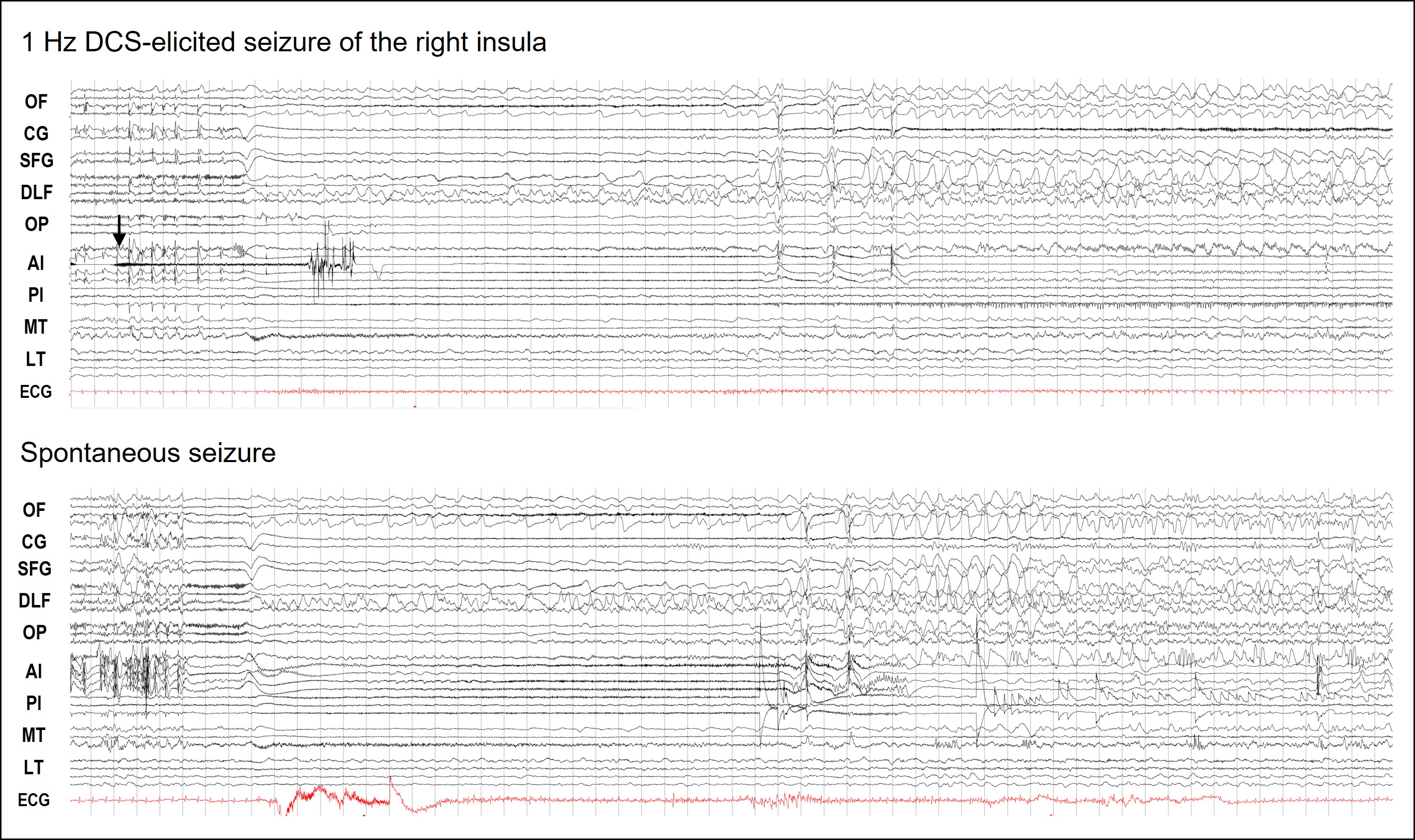

Supplement: Supplementary file 1 [file brainsci-11-01533-s001.zip › Figure S1 A.jpg]

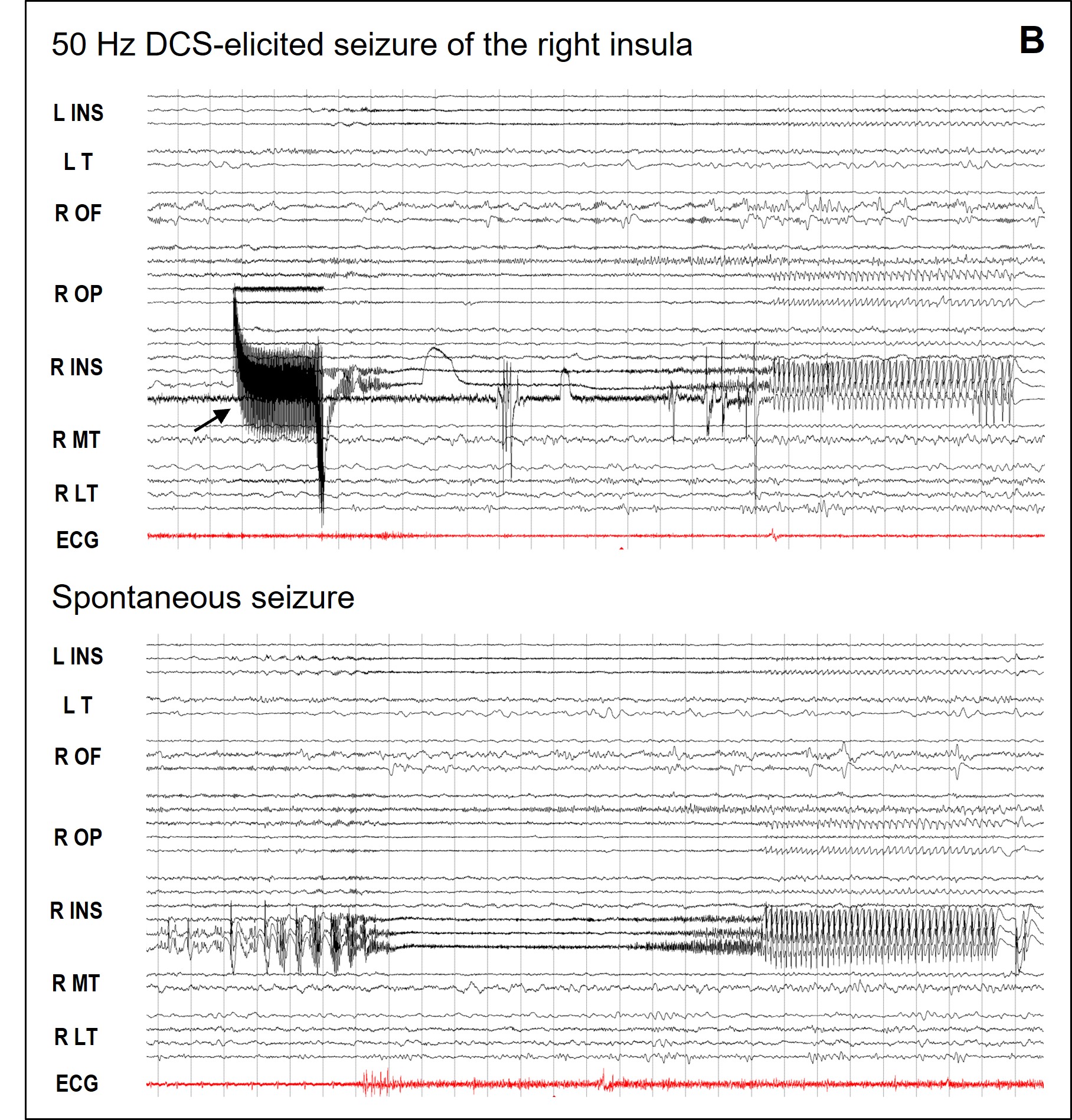

Supplement: Supplementary file 1 [file brainsci-11-01533-s001.zip › Figure S1 B.jpg]
